# Continuous Glucose Monitoring in Women with Normal OGTT in Pregnancy

**DOI:** 10.1155/2021/9987646

**Published:** 2021-08-23

**Authors:** Linda Tartaglione, Enrico di Stasio, Angelo Sirico, Mauro Di Leo, Salvatore Caputo, Alessandro Rizzi, Agnese Caneschi, Sara De Carolis, Dario Pitocco, Antonio Lanzone

**Affiliations:** ^1^Diabetes Care Unit, Department of Endocrinology and Diabetes, Catholic University of Sacred Heart, Fondazione Policlinico Universitario Agostino Gemelli IRCCS, Rome, Italy; ^2^Department of Clinical Biochemistry, Catholic University of Sacred Heart, Fondazione Policlinico Universitario Agostino Gemelli IRCCS, Rome, Italy; ^3^Department of Obstetrics and Gynecology, Catholic University of Sacred Heart, Fondazione Polclinico Universitario Agostino Gemelli IRCCS, Rome, Italy

## Abstract

Continuous glucose monitoring (CGM) might be an effective tool to improve glycemic control in gestational diabetes mellitus (GDM). Few data are available about its utilization as a diagnostic tool to find potential alterations of glycemia in subjects with normal oral glucose tolerance test (OGTT). In this preliminary prospective real-life observational study, we aimed to analyze the glycemic pattern in normal and gestational diabetes mellitus (GDM) women by continuous glucose monitoring (CGM) in order to detect potential differences between the two groups and glycemic alterations despite a normal OGTT. After the screening for GDM, subjects were connected to a CGM system for seven consecutive days. The areas under the curve of the first 60 minutes after each meal and 60 minutes before breakfast were analyzed. Women with normal OGTT that during CGM showed impaired glycemic values (more than 95 fasting or more than 140 one hour after meals or more than 120 two hours after meals) performed one week of self-monitoring of blood glucose (SMBG). After OGTT, 53 women considered normal and 46 affected by GDM were included. CGM parameters did not show any differences between the two groups with impaired glycemic excursions found in both groups. After CGM period, 33 women with normal OGTT showed abnormal glycemic patterns. These 33 women then performed one week of SMBG. After evaluation of one week of SMBG, 21 required diet therapy and 12 required insulin treatment and were followed until the delivery. An increase in gestational weight gain was observed in normal women with normal OGTT but this was not significant. No significant data were found regarding neonatal outcomes in the two groups of women. In conclusion, CGM use in pregnancy might help to detect glycemic fluctuations in women with normal OGTT, improving their treatment and outcomes.

## 1. Introduction

Gestational diabetes mellitus (GDM) is a complex widespread condition and is increasingly present in approximately 7.5-27.0% of all pregnancies [1]. It is defined by any degree of glucose intolerance recognized during pregnancy in women who do not have a previous diagnosis of diabetes [2, 3]. It represents a risk factor for short- and long-term maternal and fetal complications, including, for the mother, hypertensive disorders and delivery concerns (failure to progress in labour, caesarean section, preterm or instrumental delivery), and for the fetus, macrosomia, dystocia, neonatal hypoglycemia, and perinatal death, and for both mother and fetus, obesity, metabolic syndrome, type 2 diabetes mellitus (T2D), and cardiovascular disease [4, 5]. Macrosomia for the fetus and type 2 diabetes for the mother are the main adverse outcomes in GDM. Maternal blood glucose significantly affects fetal growth, and glycemic control is essential for adequate diabetes management [6]. Therefore, after diagnosis, patients begin a diet and exercise program, together with the self-monitoring of blood glucose (SMBG). Drug therapy is started when the recommended SMBG goals are not achieved [3]. However, there is still no agreement on GDM screening type (universal versus selective), timing, and diagnostic methods. Early pregnancy screening is recommended, but no agreement has been reached on the methods and interpretation of results between different guidelines [7, 8]. Regarding diagnosis, the current WHO statement applies the International Association of Diabetes and Pregnancy Study Groups (IADPSG) criteria [9], performing a “one-step” 75 g oral glucose tolerance test (OGTT) at 24-28 weeks of gestation, but alternative “two-step” methods are recommended by other guidelines committees [2, 10–12], by screening with a 50 g glucose load test (50 g GLT) followed by diagnosis by 100 g OGTT. The IADPSG criteria endorsed the results of the “Hyperglycemia and Adverse Pregnancy Outcomes” (HAPO) [13] study, a large-scale international cohort study involving 25505 pregnant women in nine countries. In the absence of treatment, this study shows a strong continuous relationship between any maternal glucose levels and primary outcomes, including birth weight. For the first time, glucose levels below the diabetic threshold were included in the analysis, and for most complications, no threshold for risk was found. Therefore, the debate has begun on the diagnostic-therapeutic management of those pregnant who do not fall under the GDM criteria, but belong to the category of hyperglycemia called “mild gestational diabetes,” in which the fetal maternal outcome is often adverse [14]. Furthermore, it is known that both the OGTT test at diagnosis and the self-monitoring of blood glucose during follow-up are not always reliable in terms of accuracy and reproducibility [15–17]. For these reasons, literature data suggests the use of CGM during pregnancy [18]. CGM seems superior to SMBG in detecting hypoglycemia and hyperglycemia incidents in impaired glucose tolerance and overt GDM in pregnancy, leading to more accurate decision-making during follow-up [19–21].

However, no CGM data concerning comparison between women with normal OGTT and GDM pregnant women are available. Therefore, using CGM, our aim was to compare glycemic patterns between women with normal OGTT and women with GDM diagnosed by OGTT in order to detect differences between the two groups and potential alterations of glycemia despite a normal OGTT.

## 2. Materials and Methods

### 2.1. Patients

We performed a prospective real-life observational study, recruiting a cohort of consecutive pregnant women attending our outpatient clinic from September 2018 to December 2019. Each patient was screened for GDM, which was diagnosed by OGTT between 24 and 28 weeks of pregnancy, according to the IADSPG guidelines [22]. The Italian health service uses a risk-based selective screening approach and only women with one or more risk factors for GDM (high-risk ethnicity, family history of diabetes, previous macrosomia/GDM, and advanced maternal age) were screened. Inclusion criteria were a single pregnancy and the absence of fetal malformation and/or chromosomal pathologies. The exclusion criteria were steroid treatment, previous metformin/inositol-based insulin sensitizer treatment, and forced sedentary life due to chronic neurological and/or orthopedic pathologies. The study was approved by the local ethics committee and conducted according to the principles of the Declaration of Helsinki.

All women were provided with a glucometer for measuring blood glucose on finger capillary blood until delivery. Measurements were made four times a day: in the morning after night fasting, one hour after breakfast, one hour after lunch, and one hour after dinner. The values were reported by the women either on a paper diary delivered at the time of their visit or on their smartphone, after installing the iPro2 application. The values of fasting glucose < 95 mg/dl (5.3 mmol/l) and either one-hour postprandial glucose < 140 mg/dl (7.8 mmol/l) or two-hour postprandial glucose < 120 mg/dl (6.7 mmol/l) represent the optimal control during follow-up. Each woman was also given a specific food intake of 2000 kcal, drawn up by the unit for diabetes in pregnancy, so that during the measurements, there will be a caloric and nutritional intake as homogeneous as possible between the two groups. All patients were aware of the OGTT result. The time interval between OGTT and CGM was on average one week. After OGTT, all patients (both normoglycemic and diabetic pregnant women) were submitted to CGM. All subjects during the CGM period performed SMBG as it was necessary for the calibration of the CGM. If subjects with normal OGTT showed glucose values more than 95 fasting or more than 140 one hour after meals or more than 120 two hours after meals during CGM, an intensive SMBG and diet were prescribed for at least 1 week. If during this week of SMBG, at least 20% of the values were higher than the target glucose levels (<95 mg/dl fasting, <140 mg/dl 1 hour after meal, and <120 mg/dl 2 hours after meal), insulin therapy was started. The data concerning anamnesis, anthropometric characteristics, and obstetric and neonatal outcomes have been obtained from the outpatient and inpatient medical records. Prepregnancy BMI was calculated at the first appointment before 14 gestational weeks based on the study by Fattah et al. [23], which demonstrated that there were no changes in mean maternal weight and body composition during the first trimester in a cohort of nondiabetic women. Gestational weight gain (GWG) was calculated as the difference between the maximum-recorded weight gain during pregnancy and the body weight recorded at the first visit prior to 14 weeks of gestation. The newborn population parameters evaluated were neonatal weight (NBW), neonatal weight percentile, Apgar score at 1 and 5 minutes, cordonal pH, need for admission to neonatal intensive care unit (NICU), mode, and complications of birth.

### 2.2. Continuous Glucose Monitoring

After the screening for GDM, subjects were connected to a continuous glucose monitoring (CGM) system for seven consecutive days. The women were instructed to record the time of each meal during the study period. For each meal, the area of the first 240 minutes was analyzed. Time per day within the target glucose range (TIR, between 63 and 140 mg/dl), time below the target glucose range (TBR, <63 mg/dl), and time above the target glucose range (TAR, >140 mg/dl) were also assessed (expressed in %).

We evaluated the following parameters extrapolated from the CGM: the average of all glycemic values in six days per single patient; average prebreakfast area under the curve (one hour before breakfast); average area under the postmorning curve (60minutes after breakfast); average area under the postafternoon curve (60minutes after lunch); average area under the postevening curve (60 minutes after dinner).

The glycemic monitoring used in our study is iPro™ 2 Professional Continuous Glucose Monitoring (CGM), Medtronic Minimed Inc. The instrument can detect up to 288 values in 24 hours, equal to one every 5 minutes, providing continuous, complete, and reliable glycemic profiles throughout the day. The data is collected in a CGM retrospective mode, i.e., after the woman has used the sensor, and the data were transferred by specialized medical personnel at the next check-up after 7 days using the CareLinkTM iPro™ software. Monitors were calibrated against capillary blood glucose measurements as per the manufacturer's instructions.

### 2.3. Statistical Analysis

The statistical analysis was carried out using the program “Statistical Package for Social Science (SPSS)”, version 15.0. Continuous variables are expressed as means ± standard deviation (SD) and categorical variables are represented as frequencies. The normal distribution of the data was verified using the Kolmogorov-Smirnov test. The appropriate statistical, parametric, and nonparametric test (Student's *T* or Mann-Whitney's *U* test, ANOVA, repeated measures ANOVA, Kruskal-Wallis or Friedmann's ANOVA, *χ*^2^ or Fisher test) was used for the analysis of results. All tests for statistical significance were two-sided. A *p* value of less than 0.05 indicated a significant difference.

## 3. Results

We included 46 consecutive women with diagnostic OGTT for gestational diabetes (GD) and 53 subjects with normal OGTT (Normal N).

**Figure 1:**
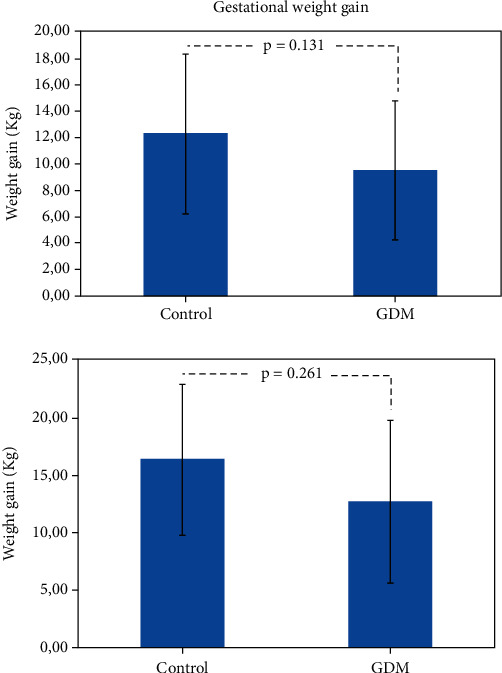
Gestational weight gain in normal women and women with gestational diabetes mellitus (GDM) expressed in Kg (a) and percentage (% (b)).

The two groups had similar characteristics in terms of age and BMI at the time of CGM positioning (Table 1); however, at first prenatal visit, a slight larger fraction of overweight/obese women was present in the GD cohort with respect to normal glucose tolerance cohort (25% vs. 36%; *p* = 0.07). Furthermore, patients in the GD group had significantly higher rates of family history of type 2 diabetes or obesity compared to the control group. Concerning gestational weight gain (GWG), the greatest increase in average weight is observed in the group of women with normal OGTT, but these differences do not reach a statistical significance (Figure 1).

Before treatment, the time interval between the evaluation of OGTT and the positioning of the CGM was on average one week.

Comparing the average daily glucose levels during CGM period in the two groups, no statistically significant differences were found (*p* = 0.145).

In all assessments, we observed that the average glucose levels were higher in group N than in the GD group, without a significant difference (Table 2).

TIR, TBR, and TAR were similar in both groups, without any significant difference (Table 2), although N showed a TAR slightly higher.

Glycemic excursions were present in both groups considering the similar food intake.

During the CGM period, were found in group N 33 women with abnormal glycemic patterns. Among these 33 women, 21 required diet therapy and 12 required insulin treatment after evaluation of one week SMBG and were followed until the delivery (Table 2).

We have subclassified normal pregnant women into 2 subgroups according to CGM results: CGM+: women with normal OGTT showing impaired glycemic control during CGM; CGM-: women with normal OGTT showing normal glycemic pattern during CGM. Significant differences were observed in plasma glucose-AUC after breakfast, time below target glucose range (<70 mg/dl), and time above target glucose range (>140 mg/dl) (Table 4 supplementary material).

The data collected after delivery showed that newborns had no major complications at birth and in the first days of life, with the exception of two cases in the GDM group: (a) one case, which presented an Apgar score of 3-6 at 1 and 5 minutes and a cordonal pH of 7.05 with an ominous neonatal outcome; it was a case with a highly premature delivery at 26 weeks; (b) a second case, who presented an Apgar score of 5-7 at 1 and 5 minutes, respectively, and a pH of 7.10, for which admission to the Neonatal Intensive Care Unit was necessary, with a following positive outcome. The majority of women (*n* = 55) delivered vaginally, 4 of which by instrumental vaginal delivery, and the remaining 44 had a caesarean section (CS). The onset of labor and the route of delivery were similar in both groups, and no differences were observed regarding the indication for CS in terms of elective CS vs. fetal concerns. There were no maternal deaths (Table 3). Finally, we compared the week of birth, the weights of newborns at the time of delivery, and the percentiles of birth weight in the two groups of women, and no statistically significant correlations were found (Table 3).

## 4. Discussion

From the criteria proposed by O'Sullivan and Mahan of 1964, gestational diabetes mellitus diagnostic criteria has evolved. While the original purpose of these criteria was primarily to assess the risk of type 2 diabetes (T2D) in the mother, subsequent studies have been designed to analyze and attempt to quantify both the possibility of adverse pregnancy and offspring outcomes [2]. The HAPO study showed that the presence of maternal hyperglycemia less severe than GDM diagnostic values is associated with an increased risk of adverse pregnancy outcomes [13]. Our study confirms the need for an improvement in the knowledge of the GDM spectrum disease. It seems that we do not have all the diagnostic tools to recognize dangerous blood sugar levels in pregnant women and the so-called “mild” gestational diabetes mellitus. A multicenter trial has shown that the treatment of mild gestational diabetes mellitus significantly reduces perinatal outcomes such as high birth weight rate, large babies for gestational age, macrosomia, and preeclampsia [24, 25]. Another study demonstrated that pregnant women, who remained untreated after negative GDM testing, developed a late-pregnancy dysglycemia related to uncontrolled weight gain which may contribute to the development of an overweight child and maternal diabetes [26]. The reproducibility and accuracy of the OGTT is questioned by some authors [15, 16]. Different studies showed that the use of fasting glycemia could be a reliable screening and diagnostic method of GDM as much as OGTT, both alone or with the postprandial plasma glucose levels [27–29]. Furthermore, a recent research showed that a single fasting plasma glucose measurement, such as OGTT, can provide a valid and predictive tool for the occurrence of unfavorable neonatal outcome [30]. Finally, a recent interesting Canadian prospective study is aimed at comparing 75 g OGTT and the SMBG in defining hyperglycemic status, and outcomes in pregnant women concluded that combining OGTT and SMBG is really effective in detecting hyperglycemic women who do not exceed GDM threshold values under OGTT alone [31]. CGM has proven to be a reliable and accurate method of glycemic control, superior to the SMBG in the recognition of episodes of hyper- and hypoglycemia during the follow-up of the GDM [18]. Law and coworkers [32] observed that GDM mothers of LGA infants have significantly higher glucose overnight compared with mothers without LGA infants. Furthermore, in pregnant women before the screening test for GDM, CGM parameters (duration and magnitude of hyperglycemic excursions measured by AUC above different thresholds) correlate with birth weight percentile [33]. In our study, CGM is applied for the first time immediately after the OGTT execution in order to detect impaired glucose levels (more than 95 fasting or more than 140 one hour after meals or more than 120 two hours after meals) despite a normal OGTT. CGM showing the blood glucose patterns after the meals mimics what happens during a mixed-meal tolerance test is as effective as the OGTT in diagnosing impaired glucose tolerance and is even more sensitive [34]. Interestingly, our data highlight the lack of difference in the percentage of TBR between normal and GDM pregnant women, according to literature data [35]. In our opinion, the absence of differences between subjects with gestational diabetes diagnosed through OGTT and pregnant women with normal OGTT is due to the greater CGM ability to detect glycemic excursions. After the subclassification of women with normal OGTT, we did not find significant differences about risk factors for gestational diabetes in these two subgroups although this analysis might be limited by the small sample size. These results can further support the relevance of weight gain in the pathogenesis of glycemic fluctuations during the pregnancy. Concerning the pattern found during CGM, it should be underlined that there was a significant difference between these two subgroups especially in the area under the curve 1 hour postbreakfast. This could mean that some particular dietary modifications such as the utilization of a breakfast with low glycemic index could be useful to prevent it, for example, when an important weight gain is present.

The absence of differences in neonatal outcomes is probably also due to the management of patients led by CGM, which allowed a more aggressive approach on a subject considered nondiabetic after OGTT. Moreover, an interesting result of our study, although not significant, is the increase in body weight of subjects considered nondiabetic after OGTT. The trend of an increased weight gain in the group with normal OGTT could be linked to the lack or delay in the offering of focused lifestyle counseling for the group not known to have GDM, compared to the group known to have GDM after OGTT. According to the real-life study design, the patients were aware of the GDM diagnosis based on OGTT, and therefore, they may have paid attention to diet more strictly than the group with normal results. On the other hand, the 2009 Institute of Medicine (IOM) guidelines have different recommendations on gestational weight gain for overweight and normal-weight women that could also explain the findings of a slightly greater gain in weight in normal women than GDM women in the present manuscript [35].

We cannot rule out that the impaired glycemic values of many of the subjects during CGM is the consequence of the gestational weight gain (GWG). The HAPO study showed that maternal BMI is an independent risk factor for maternal blood glucose levels [13]. A recent paper confirms these data and showed that excessive gestational weight gain (eGWG) is a “synergic risk factor” for poor outcome in both obesity and in GDM [36, 37]. However, a study published by Kong et al. [38] concluded that maternal diabetes under insulin treatment appears to be associated with a marked risk of LGA and preterm birth, while maternal obesity associated with type 2 diabetes has only a moderately increased risk. In the study design, however, the authors did not consider the weight gain that the enrolled women had during pregnancy and its important role in fetal macrosomia [39, 40].

Our data seem large enough to suggest that the management of glucose levels, after CGM results, makes the 2 groups, controls and GDM subjects, completely similar for fetal outcomes. Concerning this field, our data seem to confirm recent publications on this topic that did not show differences in fetal growth and birth weight percentiles of neonates born to GDM mothers (classified as medium or low risk) and NGT women [41, 42].

The fact that 44% of our patients needed a cesarean is quite high and requires an explanation. The high rate of cesarean delivery may be associated with the increased CS rate found in untreated mild hyperglycemia, as in the HAPO study, and with the known increased CS rate in our country [43]. Limitations: our study undoubtedly has limitations, the greatest of which is the low number of participants and the lack of data concerning hypertensive disorders. For this reason, a subclassification between normal subjects and subjects with impaired glycemia after CGM has not been included in the paper. We do not think the results of this study lead to actionable conclusions without further substantial analysis or additional studies and the consideration of the feasibility of actually doing CGM on a large scale for pregnant women. Realistically, in order to avoid excessive medicalization of pregnancy, the widespread use of technology requires more robust evidence, and therefore, a long-term large controlled clinical trial on this topic is mandatory.

## 5. Conclusion

The diagnosis of gestational diabetes mellitus based on current WHO criteria could be insufficient to identify all pregnant women with abnormal glycemic excursions although it remains the chosen tool. The addition of a CGM period could be a good tool to detect glycemic fluctuations and improve the management of these patients. We are aware that, currently, CGM cannot replace OGTT as diagnostic tool, especially from a cost-effective point of view. However, in the future, a holistic approach to mild GDM, through the use of continuous glucose monitoring, probably as an integral part of a metabolic gestational score involving maternal and fetal anthropometric parameters could really distinguish which pregnant women should be followed by the caregivers in terms of more intensive management, to counteract the short- and long-term maternal and fetal complications.

## Figures and Tables

**Table 1:** Baseline features in normal women and women with gestational diabetes mellitus (GDM); BMI: body mass index; DM: diabetes mellitus; OGTT: oral glucose tolerance test; Significant *p* values are in bold.

	Normal (*n* = 53)	GDM (*n* = 46)	*p*
Mean age of mothers (yrs.)	34 ± 5	33 ± 6	0.528
BMI (kg/m^2^)	24.0 ± 3.3	24.7 ± 3.1	0.253
Smokers *n* (%)	2 (3.6%)	2 (3.7%)	0.628
Family history *n* (%) for type 2 DM/obesity	9 (17%)	25 (54.3%)	**<0.001**
Plasma glucose (OGTT 0') (mg/dl)	79 ± 7	79 ± 5	0.930
Plasma glucose (OGTT 60') (mg/dl)	130 ± 23	186 ± 26	**<0.001**
Plasma glucose (OGTT 120') (mg/dl)	116 ± 20	171 ± 29	**<0.001**
HbA1C			
%	4.8 ± 0.49	5.2 ± 0.51	0.114
Mmol/Mol	29 ± 2.9	33 ± 3.2	

**Table 2:** Continuous glucose monitoring (CGM) parameters and treatments in normal women and women with gestational diabetes mellitus (GDM). TIR: time per day within target glucose range (between 70 and 140 mg/dl); TBR: time below target glucose range (<70 mg/dl); TAR: time above target glucose range (>140 mg/dl); AUC: area under the curve; Significant *p* values are in bold.

	Normal (*n* = 53)	GDM (*n* = 46)	*p*
Mean plasma glucose (mg/dl) (all values in six days)	98 ± 9	95 ± 8	0.145
Plasma glucose-AUC before breakfast (mg/dl/min.)	6623 ± 771	6400 ± 1003	0.223
Plasma glucose-AUC after breakfast(mg/dl/min.)	7277 ± 1096	7073 ± 1405	0.427
Plasma glucose-AUC after lunch (mg/dl/min.)	7444 ± 1183	7241 ± 1389	0.439
Plasma glucose-AUC after dinner (mg/dl/min.)	7481 ± 1510	7145 ± 1460	0.263
TBR (time <63 mg/dl, %)	5.3 ± 5.3	7.6 ± 8.0	0.091
TIR (time 63-140 mg/dl, %)	89.6 ± 5.2	88.8 ± 7.6	0.534
TAR (time >140 mg/dl, %)	5.1 ± 4.6	3.6 ± 3.0	0.065
New impaired glycemic control women, *n* (%)	33 (62%)	—	
*Treatment*			
Nutritional therapy, *n* (%)	21 (39.0%)	46 (100%)	**p** ≤ 0.001
Insulin, *n* (%)	12 (22.6%)	35 (76.1%)	**p** ≤ 0.001

**Table 3:** Delivery features and neonatal outcomes in normal women and women with gestational diabetes mellitus (GDM).

	Normal (*n* = 53)	GDM (*n* = 46)	*p*
Gestational age at delivery (yrs.)	37.7 ± 5	37.8 ± 3	0.913
Delivery modality			
Vaginal, *n* (%)	27 (51%)	28 (61%)	0.321
Caesarean, *n* (%)	26 (49%)	18 (39%)	0.236
Weight (kg)	3147 ± 891	2951 ± 710	0.498
Weight percentile	56.2 ± 23	51.1 ± 30	0.514
Large weight for gestational age, *n* (%)	—	2 (4.6%)	0.539
Small weight for gestational age, *n* (%)	4 (7.5%)	2 (4.6%)	0.821

## Data Availability

A reformulation of our data statement is not necessary and data ara available upon reasonable request to the authors.

## Supplementary Materials

Supplementary Materials This file includes Table 4, in which we provide the subclassification between normal glucose tolerant women and those with impaired glycemia after CGM. We did not find significant differences about risk factors for gestational diabetes in these two subgroups although this analysis might be limited by the small sample size. These results can further support the relevance of weight gain in the pathogenesis of glycemic fluctuations during the pregnancy. Concerning the pattern found during CGM, it should be underlined that there was a significant difference between these two subgroups especially in the area under the curve 1 hour postbreakfast. It might mean that some particular dietary modifications to prevent it could be useful, for example, the utilization of a breakfast with low glycemic index, in particular, when an important weight gain is present.
