# Supplementary material for: Continuous Glucose Monitoring in Women with Normal OGTT in Pregnancy
Source: J Diabetes Res. 2021 Aug 23;2021:9987646. doi: 10.1155/2021/9987646 (PMC8408006; doi:10.1155/2021/9987646)
Supplement: Supplementary Materials — This file includes Table 4, in which we provide the subclassification between normal glucose tolerant women and those with impaired glycemia after CGM. We did not find significant differences about risk factors for gestational diabetes in these two subgroups although this analysis might be limited by the small sample size. These results can further support the relevance of weight gain in the pathogenesis of glycemic fluctuations during the pregnancy. Concerning the pattern found during CGM, it should be underlined that there was a significant difference between these two subgroups especially in the area under the curve 1 hour postbreakfast. It might mean that some particular dietary modifications to prevent it could be useful, for example, the utilization of a breakfast with low glycemic index, in particular, when an important weight gain is present. [file 9987646.f1.docx]

|  | Normal  (n=53) | CGM-  (n=20) | CGM+  (n=33) | p |
| --- | --- | --- | --- | --- |
|  |  |  |  |  |
| Mean age of mothers (yrs.) | 34 ± 5 | 33 ± 5 | 33 ± 8 | 0.806 |
| BMI (Kg/m^2^) | 24.0 ± 3.3 | 24.9 ± 4.4 | 23.4 ± 2.8 | 0.164 |
| Family history n (%)  for type 2 DM/Obesity | 9/53 (17%) | 4/20 (20%) | 5/33 (15.1%) | 0.715 |
| Plasma glucose (OGTT 0’) (mg/dl) | 79 ± 7 | 80 ± 7 | 79 ± 7 | 0.446 |
| Plasma glucose (OGTT 60’) (mg/dl) | 130 ± 23 | 128 ± 24 | 130 ± 23 | 0.716 |
| Plasma glucose (OGTT 120’) (mg/dl) | 116 ± 20 | 112 ± 20 | 118 ± 21 | 0.309 |
| HbA1C  %  Mmol/mol | 4.8 ± 0.49  29 ± 2.9 | 4.7 ± 0.53  27.9 ± 3.1 | 4.8 ± 0.52  29 ± 3.1 | 0.860 |
| Mean plasma glucose (mg/dl)  (all values in six days) | 98 ± 9 | 96 ± 9 | 99 ± 9 | 0.308 |
| Plasma glucose-AUC before breakfast (mg/dl/min.) | 6623 ± 771 | 6528 ± 650 | 6680 ± 839 | 0.464 |
| Plasma glucose-AUC after breakfast(mg/dl/min.) | 7277 ± 1096 | 6859 ± 827 | 7530 ± 1171 | **0.018** |
| Plasma glucose-AUC after lunch (mg/dl/min.) | 7444 ± 1183 | 7077 ± 1174 | 7666 ± 1149 | 0.082 |
| Plasma glucose-AUC after dinner (mg/dl/min.) | 7481 ± 1510 | 7310 ± 1755 | 7584 ± 1358 | 0.554 |
| TBR (Time < 70 mg/dl, %) | 5.3 ± 5.3 | 7.7 ± 6.8 | 3.7 ± 3.3 | **0.023** |
| TIR (Time 70 -140 mg/dl, %) | 89.6 ± 5.2 | 91.2 ± 6.5 | 88.6 ± 3.9 | 0.113 |
| TAR (Time > 140 mg/dl, %) | 5.1 ± 4.6 | 1 ± 0.9 | 7.6 ± 4.1 | **0.000** |

Table 4: main baseline and continuous glucose monitoring (CGM) parameters in normal women subclassified according to CGM results: CGM + = women with normal OGTT showing impaired glycemic control during CGM; CGM - = women with normal OGTT showing normal glycemic pattern during CGM. BMI=body mass index; DM= diabetes mellitus; OGTT=oral glucose tolerance test. TIR = Time per day within target glucose range (between 70 and 140 mg/dl); TBR = Time below target glucose range (< 70 mg/dl); TAR = Time above target glucose range (> 140 mg/dl); AUC=area under the curve.
